# Partial least squares enhance multi-trait genomic prediction of potato cultivars in new environments

**DOI:** 10.1038/s41598-023-37169-y

**Published:** 2023-06-19

**Authors:** Rodomiro Ortiz, Fredrik Reslow, Abelardo Montesinos-López, José Huicho, Paulino Pérez-Rodríguez, Osval A. Montesinos-López, José Crossa

**Affiliations:** 1 Department of Plant Breeding, Swedish University of Agricultural Sciences (SLU), P.O. Box 190, SE 23436 Lomma, Sweden; 2 Departamento de Matemáticas, Centro Universitario de Ciencias Exactas e Ingenierías (CUCEI), 44430 Guadalajara, México; 3 International Maize and Wheat Improvement Center (CIMMYT), Carretera México-Veracruz Km. 45, El Batán, 56237 Texcoco, Edo. de México México; 4 Colegio de Postgraduados (COLPOS), 56230 Montecillos, Edo. de México México; 5 Facultad de Telemática, Universidad de Colima, 28040 Colima, México; 6 Centre for Crop and Food Innovation, Food Futures Institute, Murdoch University, Murdoch, Australia

**Keywords:** Genetics, Heritable quantitative trait, Quantitative trait loci

## Abstract

It is of paramount importance in plant breeding to have methods dealing with large numbers of predictor variables and few sample observations, as well as efficient methods for dealing with high correlation in predictors and measured traits. This paper explores in terms of prediction performance the partial least squares (PLS) method under single-trait (ST) and multi-trait (MT) prediction of potato traits. The first prediction was for tested lines in tested environments under a five-fold cross-validation (5FCV) strategy and the second prediction was for tested lines in untested environments (herein denoted as leave one environment out cross validation, LOEO). There was a good performance in terms of predictions (with accuracy mostly > 0.5 for Pearson’s correlation) the accuracy of 5FCV was better than LOEO. Hence, we have empirical evidence that the ST and MT PLS framework is a very valuable tool for prediction in the context of potato breeding data.

Potato (*Solanum tuberosum* L.) ranks third among food crops in human diets. The most widely grown potatoes are self-compatible, polysomic tetraploid species (2*n* = 4*x* = 48 chromosomes) with tetrasomic inheritance and inbreeding depression after selfing. Potato cultivars or breeding clones are often highly heterozygous, and tuber yield benefits from heterosis. Potato is a vegetatively propagated crop in which each tuber is identical to its mother plant, thus allowing favorable traits to be fixed in the hybrid. Tuber yield is a quantitative trait of multi-genic structure, thus making it difficult to evaluate in the early stages of potato breeding^1^.

The main objective in potato breeding is increasing productivity and quality as well as resilience in stressful environments. However, tuber yield gains are stagnated^2,3^. Ortiz et al.^4^ estimated that annual productivity gains in European potato cultivars were 0.7% in the last 60 years while the yearly genetic gains for tuber yield considering only cultivars released after the 2nd World War were about 0.36%. Annual genetic gains for breeding low reducing sugars in the tuber flesh, and high host plant resistance to late blight were below 0.2%. Based on the low genetic gains for traits, it is important to revamp today’s potato crossbreeding schemes using a modern approach like genomic prediction for selection (hereinafter genomic selection) of promising germplasm. The market preferences regarding potato uses may slow adopting new cultivar, thus resulting in low genetic gains over time.

Genomic selection (GS) is a methodology that uses statistical models and training data to improve the selection early in time without the need to measure phenotypic information^5^. The success of GS methodology are known in crops such as cassava, chickpea, groundnut, maize, rice, or wheat^6–9^. There are several components affecting the prediction performance of GS methodology. Some are related to optimizing the design of the training set whereas other factors are related to the quality of the marker data, and the relationship training–testing sets^10^. The important challenge of GS is its practical implementation in assisted breeding because GS methodology does not always guarantee medium or high prediction accuracy of the unobserved cultivars.

Selecting the best statistical and machine learning tools for GS implementation are a high research priority. Applications in GS prediction using random forest, mixed models, Bayesian methods, support vector machine, gradient boosting machine methods and deep learning methods are available. However, none of these models, methods or algorithms are the best statistical machine learning methods for predictive modeling problems such as classification and regression^11^. There are specific cases where a particular algorithm consistently outperformed another. For example, there is empirical evidence that the deep neural network method, when image analysis is incorporated into the GS prediction, performed better than other models. Yet a limitation of deep neural network models is the need for both large datasets and very intense computing capacity^12–14^.

Genomic selection can predict performance in future seasons or new locations because it is based on genomic prediction models, which is an important factor in the success of plant breeding. This is the main reason why standard genomic prediction (GP) models were extended to multi-environment prediction by modeling genotype × environment interactions (GE) using linear mixed random effects models, in which the main effects of markers and environmental covariates could be introduced using covariance structures that are functions of marker genotypes and environments^15,16^. Cuevas et al.^17^ and Sousa et al.^18^ applied the marker × environment interaction GS model of Lopez-Cruz et al.^19^ and modeled GE through a non-linear Gaussian kernel. These authors concluded that the higher prediction accuracy of models including GE and Gaussian kernel is due to accounting for more complex marker main effects and marker-specific interaction effects.

Potato breeding must improve its efficiency by increasing the reliability of selection as well as identifying promising germplasm for crossing. Ortiz et al.^20^ investigated the genomic prediction accuracy of estimated breeding values for several potato breeding clones and cultivars for three dosages of marker alleles [pseudo-diploid (A); additive tetrasomy polyploidy (B); additive-non-additive tetrasomy polyploidy (C)] for a single environment and multiple-environments accounting for GE, and comparing two kernels, the linear Genomic Best Linear Unbiased Predictor (GBLUP) and the non-linear Gaussian kernel (GK) when used with the single-kernel genetic combined matrices of A, B, C or when employing two-kernel genetic matrices B and C for a single environment and for multi-environments modeling GE. Multi-environment (ME) modeling had prediction accuracy estimates higher than those obtained from the single-environment (SE) analyses. GBLUP was the best method in combination with the markers structure B for predicting most of the tuber traits. Most of the potato traits gave relatively high prediction accuracy under this combination of marker structure (A, B, C, and B-C) and methods GBLUP and GK combined with the ME model that considers the GE.

Research shows that the parametric Bayesian methods incorporating genomic (G) and GE (by means of GBLUP) are robust enough for producing competitive predictions without the need to invest extra time for the tuning process as well as the obvious advantage regarding other statistical learning machine methods that require no effort to select the hyperparameters. Assessing the GE by the non-linear Gaussian kernel is often a better option for modeling GE than the linear kernel GBLUP^7^.

For the prediction of new environments (or seasons), most statistical machine learning methods have difficulty achieving reasonable prediction accuracy, because it depends on the relationship between individuals in training/testing, sample size, marker density, or GE, among others. For this reason, the prediction of a new season or environments is a more challenging task that when using known strategies of cross-validation. In multi-environmental plant breeding field trials, information on environments may enhance the information in GE. Aastveit and Martens^21^ proposed the partial least squares (PLS) regression method to describe GE in terms of differential sensitivity of cultivars to environmental variables, in which explanatory variables are linear combinations of the complete set of measured environmental or cultivar variables with no limit to the number of exploratory covariables. Based on the above considerations, Montesinos et al. explored PLS for the prediction of new environments, by leaving one environment out (LOEO). Montesinos-López et al.^22^ also compared the single unit-trait (ST) PLS (ST-PLS) prediction accuracy with that of the ST-GBLUP and showed clear empirical evidence of the power of PLS methodology for the prediction of future seasons or new environments. Montesinos-López et al.^23^ proposed an improved Bayesian multi-trait (MT) and ME (BMTME) R package that implements the BMTME model^24^ and can capture the correlation not only between lines, but also between traits and environments. With the continuous and dramatic growth of computational power, MT models play an increasingly important role in the statistical learning methods for selecting the best predictive model.

The use of MT models is not as widespread as ST models because fitting MT models is more computationally demanding than fitting ST models, and has more complex GE since traits have different response patterns in different environments. MT models have more problems of convergence than ST models, and implementing MT models for genomic prediction is more challenging due to the size and complexity of the underlying data sets^25^. However, in a recent potato study on GS prediction, Cuevas et al.^26^ investigated ST versus MT in ME models for the combination of six environments for five tuber weight traits and two tuber flesh quality characteristics. The best predictive model was the MT for predicting several traits of potato observed in some environments and predicted in other environments.

The Multi-Trait Partial Least Square (MT-PLS) regression can model complex biological events, and research suggests that the MT-PLS is a potentially valuable method for modeling high-dimensional biological data^27^. MT-PLS can model multiple responses, while efficiently dealing with multicollinearity. Joint association analysis like MT-PLS explicitly uses the correlation structure among traits and thus it is preferred over ST-PLS. In a recent study, Montesinos et al.^28^ found an increase in prediction accuracy of MT-PLS over the MT-GBLUP.

Based on the previous knowledge and the need to investigate different options of genomic prediction models that will consider the important breeding task of predicting future location-year combinations, the main objective of this study was to predict unobserved potato cultivars by means of MT-PLS and ST-PLS. In this research we used potato breeding trial datasets comprising the combination of three locations in Sweden where up to 256 potato cultivars and breeding clones were tested during 2 years for tuber weight traits and tuber flesh quality characteristics.

## Results

**Figure 1:**
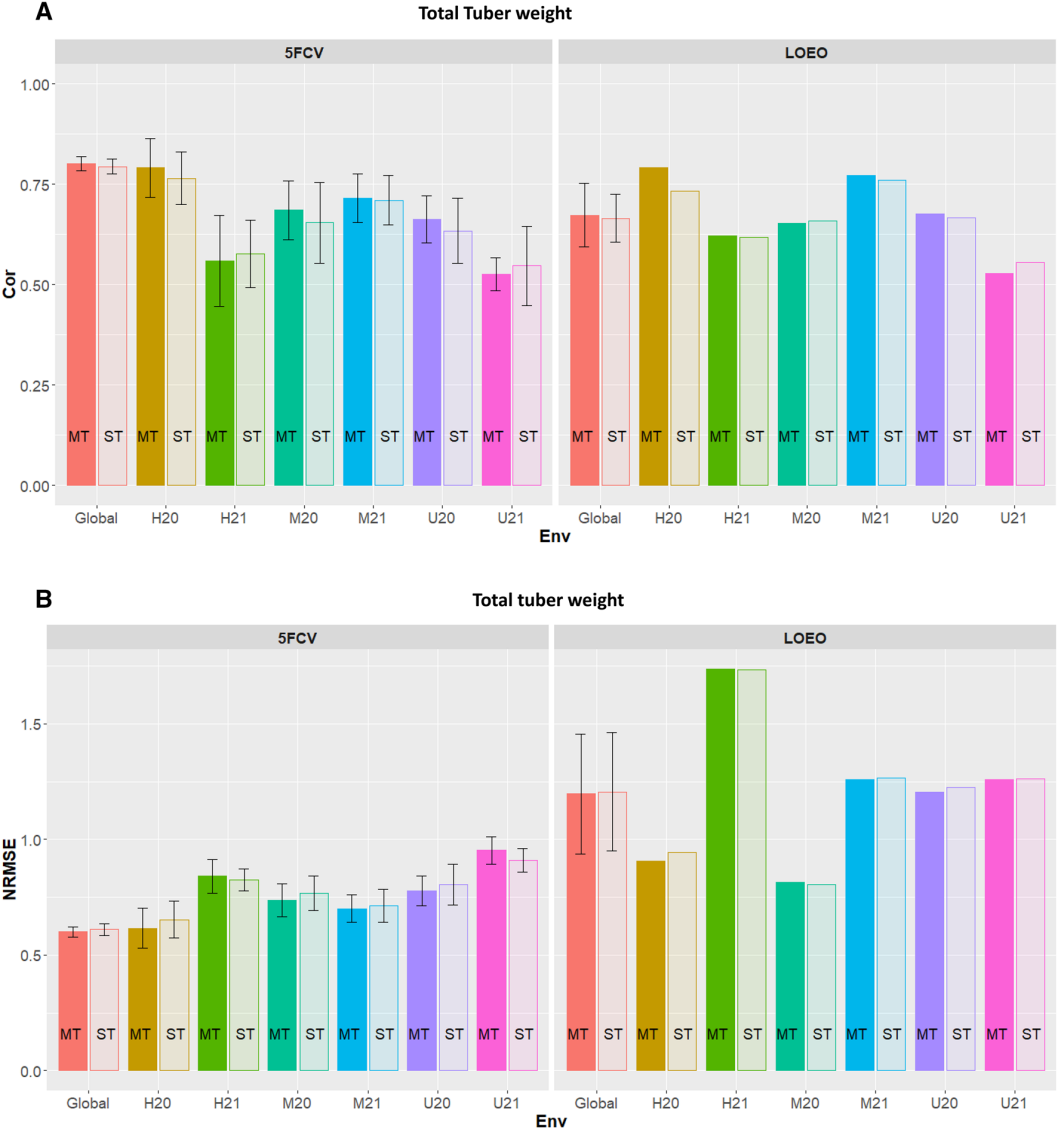
Trait Total tuber weight. (**A**) Correlation (Cor) between observed and predicted values for Multi-trait (MT) and single trait (ST) for fivefold cross-validation (5FCV) and leave-one-environment-out (LOEO) for each location-year combination (*H* Helgegården, *M* Mosslunda, *U* Umeå; *20* Year 2020, *21* Year 2021, *Global* across six environments). (**B**) Normalized Root Mean Squared Error (NRMSE) for MT and ST for 5FCV and LOEO for each location-year combination (*H* Helgegården, *M* Mosslunda, *U* Umeå; *20* Year 2020, *21* Year 2021, *Global* across six environments).

**Figure 2:**
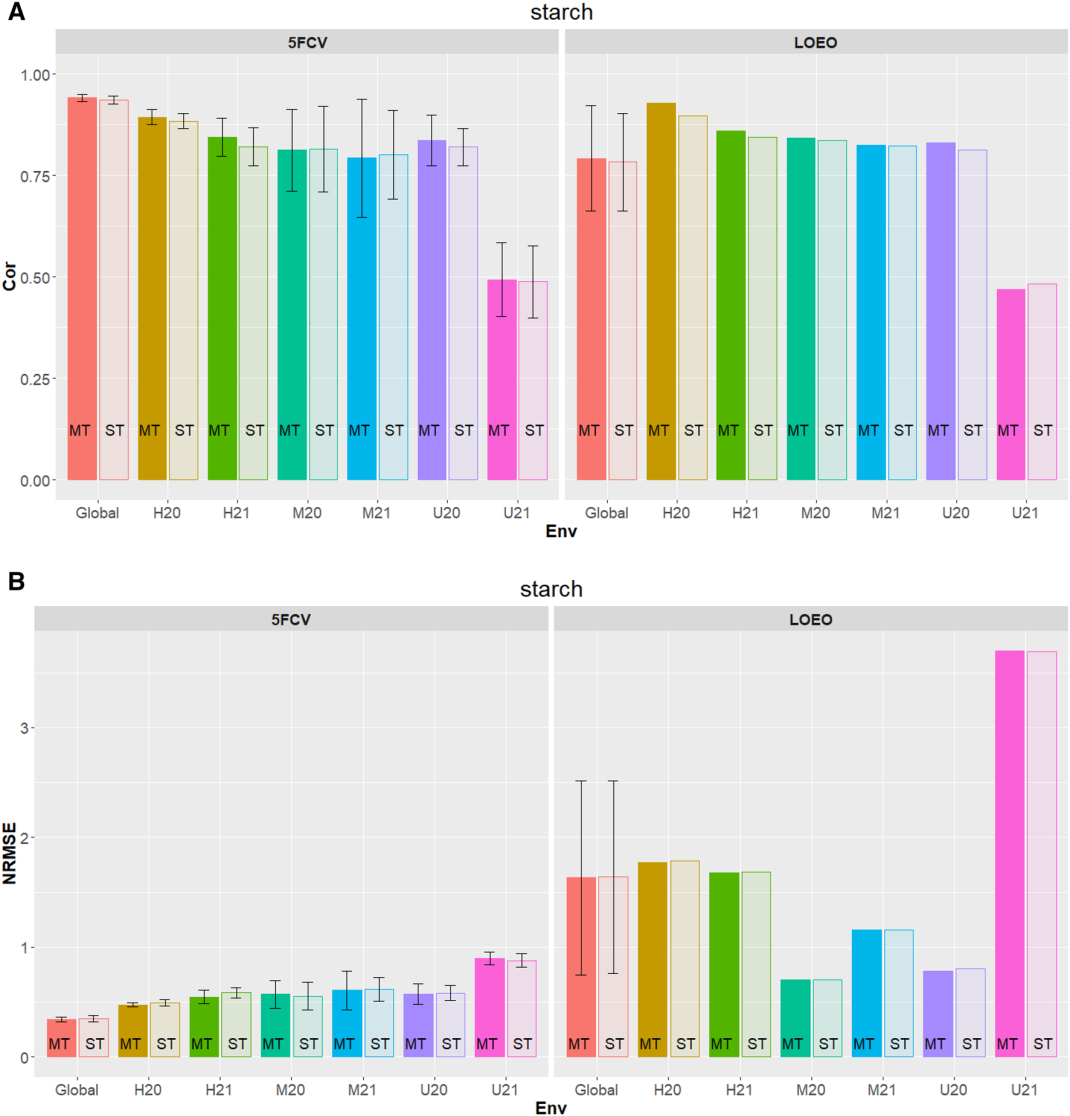
Starch (%) in the tuber flesh. (**A**) Correlation (Cor) between observed and predicted values for Multi-trait (MT) and single trait (ST) for fivefold cross-validation (5FCV) and leave-one-environment-out (LOEO) for each location-year combination (*H* Helgegården, *M* Mosslunda, *U* Umeå; *20* Year 2020, *21* Year 2021, *Global* across six environments). (**B**) Normalized Root Mean Squared Error (NRMSE) for MT and ST for 5FCV and LOEO for each location-year combination (*H* Helgegården, *M* Mosslunda, *U* Umeå; *20* Year 2020, *21* Year 2021, *Global* across six environments).

**Figure 3:**
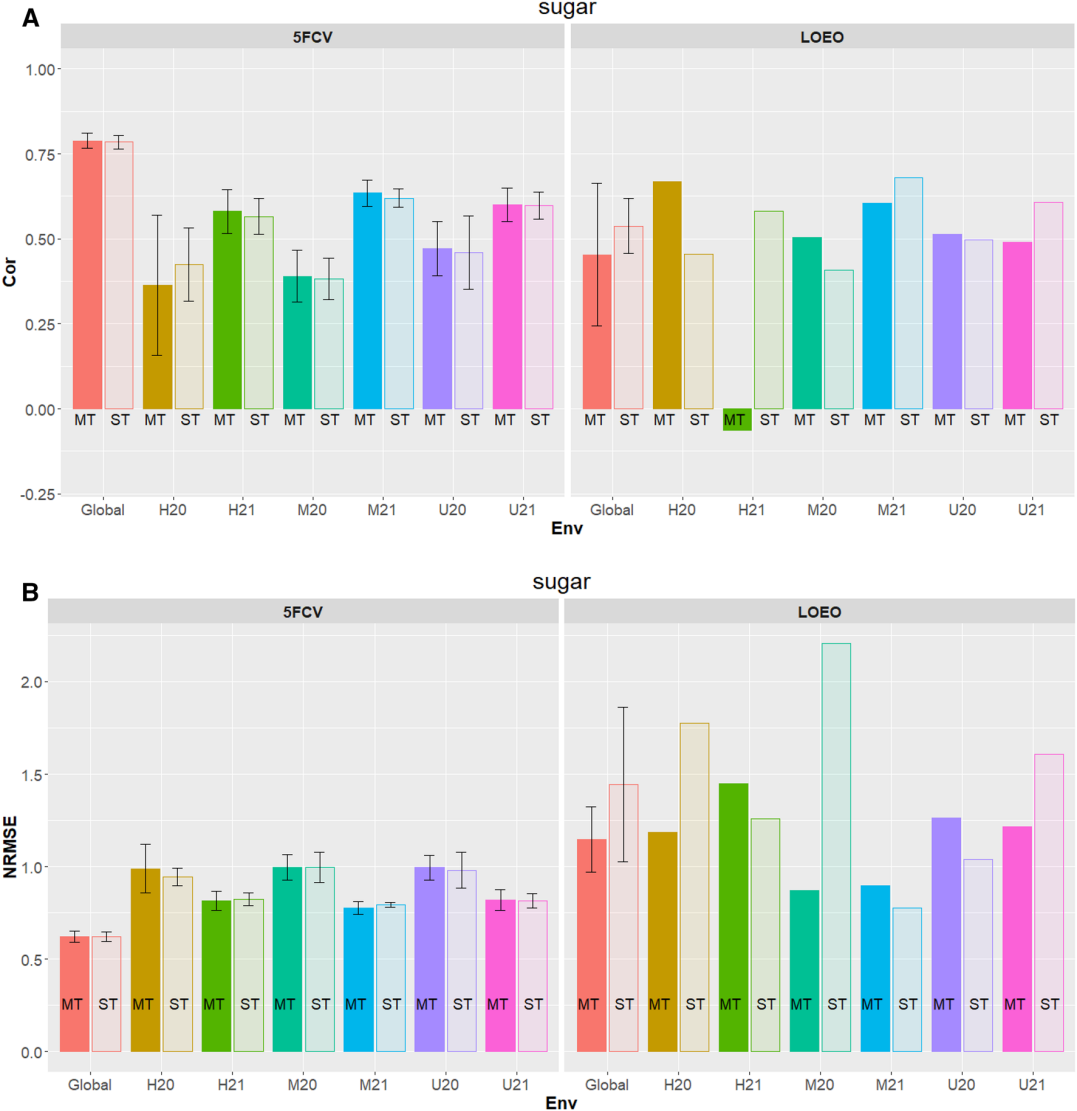
Reducing sugars in the tuber flesh. (**A**) Correlation (Cor) between observed and predicted values for Multi-trait (MT) and single trait (ST) for fivefold cross-validation (5FCV) and leave-one-environment-out (LOEO) for each location-year combination (*H* Helgegården, *M* Mosslunda, *U* Umeå; *20* Year 2020, *21* Year 2021, *Global* across six environments). (**B**) Normalized Root Mean Squared Error (NRMSE) for MT and ST for 5FCV) and LOEO for each location-year combination (*H* Helgegården, *M* Mosslunda, *U* Umeå; *20* Year 2020, *21* Year 2021, *Global* across six environments).

Tables and figures are shown for two metrics: (1) correlations (ρ) between predictive genetic values and their corresponding observed values (testing set) and (2) the normalized root mean squared error of prediction (NRMSE) for single-trait ST-PLS and multi-trait MT-PLS under two cross-validation, 5FCV and LOEO (including their standard errors, SE when appropriate). Tables 1, 2 and 3 list the prediction accuracy results for three traits, total tuber weights, flesh tuber starch (%) and flesh reducing sugar, respectively. Also, the results from Tables 1, 2 and 3 are displayed in Figs. 1, 2 and 3, respectively. Supplementary Table S1 and S2 provide heritability estimates based on variance components, and genetic/phenotypic correlations, respectively. Furthermore, results of genomic prediction accuracy for metrics correlation and NRMSE for tuber weight according to their tuber size (< 40 mm, 40–50 mm, 50–60 mm and > 60 mm) are given in Supplementary Figs. S1–S4. Supplementary Tables S1–S4 give the prediction accuracy results for ST-PLS, MT-PLS, and NRMSE for 5FCV, and LOEO of these traits.

**Table 1:** Partial least square (PLS) accuracy measured as correlation between observed and predicted values (ρ), and normalized Root Mean Squared Error (NRMSE) with their respective standard errors (SE) for genomic prediction of total tuber weight in 10-plant plots considering single trait PLS (ST-PLS)- and multi-trait PLS (MT-PLS) for each location within in each year and across environments considering five-fold random cross-validations (5FCV) and leaving one environment out (LOEO).

Environment	ρ	SE	NRMSE	SE	ρ	SE	NRMSE	SE
	ST-PLS				MT-PLS			
5FCV								
HEL20	0.7651	0.0336	0.6532	0.0410	0.7910	0.0370	0.6163	0.0437
HEL21	0.5761	0.0429	0.8253	0.0239	0.5592	0.0575	0.8417	0.0373
MOS20	0.6549	0.0513	0.7672	0.0374	0.6858	0.0375	0.7372	0.0368
MOS21	0.7107	0.0313	0.7144	0.0362	0.7160	0.0308	0.7008	0.0306
UM20	0.6341	0.0414	0.8041	0.0447	0.6625	0.0296	0.7773	0.0328
UM21	0.5471	0.0502	0.9098	0.0266	0.5260	0.0213	0.9526	0.0308
Across	0.7946	0.0093	0.6099	0.0131	**0.8017**	0.0089	0.6000	0.0112
LOEO								
HEL20	0.7340	–	0.9444	–	0.7910	–	0.9065	–
HEL21	0.6189	–	1.7354	–	0.6221	–	1.7359	–
MOS20	0.6590	–	0.8050	–	0.6523	–	0.8155	–
MOS21	0.7599	–	1.2648	–	0.7713	–	1.2581	–
UM20	0.6671	–	1.2245	–	0.6770	–	1.2045	–
UM21	0.5548	–	1.2627	–	0.5279	–	1.2599	–
Across	0.6656	0.0306	1.2061	0.1312	0.6736	0.0399	1.1967	0.1327

The largest ρ in bold font.

*HEL* Helgegården, *MOS* Mosslunda, *UM* Umeå, *20* Year 2020, *21* Year 2021, *Global* across six environments.

**Table 2:** Partial least square (PLS) accuracy measured as correlation between observed and predicted values (ρ), and normalized Root Mean Squared Error (NRMSE) with their respective standard errors (SE) for genomic prediction of flesh tuber starch (%)considering single trait PLS (ST-PLS)- and multi-trait PLS (MT-PLS) for each location within each year and across environments considering five-fold random cross-validations (5FCV) and leaving one environment out (LOEO).

Environment	ρ	S.E	NRMSE	S.E	ρ	S.E	NRMSE	S.E
	ST-PLS				MT-PLS			
5FCV								
HEL20	0.8845	0.0092	0.4922	0.0139	0.8938	0.0096	0.4738	0.0087
HEL21	0.8209	0.0238	0.5843	0.0246	0.8444	0.0236	0.5472	0.0304
MOS20	0.8152	0.0540	0.5531	0.0641	0.8126	0.0515	0.5699	0.0643
MOS21	0.8012	0.0555	0.6152	0.0556	0.7932	0.0741	0.6057	0.0911
UM20	0.8204	0.0232	0.5835	0.0348	0.8367	0.0315	0.5722	0.0486
UM21	0.4883	0.0451	0.8789	0.0306	0.4939	0.0460	0.8985	0.0309
Across	0.9367	0.0052	0.3502	0.0141	**0.9413**	0.0045	0.3433	0.0111
LOEO								
HEL20	0.8974	–	1.7861	–	0.9279	–	1.7750	–
HEL21	0.8452	–	1.6820	–	0.8602	–	1.6745	–
MOS20	0.8359	–	0.7065	–	0.8421	–	0.7032	–
MOS21	0.8231	–	1.1575	–	0.8242	–	1.1589	–
UM20	0.8130	–	0.8017	–	0.8308	–	0.7833	–
UM21	0.4831	–	3.6949	–	0.4699	–	3.6963	–
Across	0.7830	0.0612	1.6381	0.4492	0.7925	0.0663	1.6319	0.4506

The largest ρ in bold font.

*HEL* Helgegården, *MOS* Mosslunda, *UM* Umeå, *20* Year 2020, *21* Year 2021, *Global* across six environments.

**Table 3:** Partial least square (PLS) accuracy measured as correlation between observed and predicted values (ρ), and normalized Root Mean Squared Error (NRMSE) with their respective standard errors (SE) for genomic prediction of flesh reducing sugars considering single trait PLS (ST-PLS)- and multi-trait PLS (MT-PLS) for each location within each year and across environments considering five-fold random cross-validations (5FCV) and leaving one environment out (LOEO).

Environment	ρ	S.E	NRMSE	S.E	ρ	S.E	NRMSE	S.E
	ST-PLS				MT-PLS			
5FCV								
HEL20	0.4247	0.0549	0.9451	0.0236	0.3635	0.1055	0.9896	0.0671
HEL21	0.5661	0.0270	0.8239	0.0174	0.5806	0.0329	0.8153	0.0255
MOS20	0.3826	0.0308	0.9969	0.0412	0.3903	0.0394	0.9955	0.0354
MOS21	0.6199	0.0139	0.7937	0.0065	0.6346	0.0195	0.7777	0.0180
UM20	0.4604	0.0550	0.9805	0.0490	0.4707	0.0408	0.9950	0.0344
UM21	0.5984	0.0204	0.8154	0.0194	0.6002	0.0247	0.8196	0.0282
Across	0.7843	0.0105	0.6204	0.0130	**0.7887**	0.0112	0.6224	0.0151
LOEO								
HEL20	0.4557	–	1.7767	–	0.6691	–	1.1876	–
HEL21	0.5811	–	1.2577	–	− 0.0624	–	1.4475	–
MOS20	0.4075	–	2.2048	–	0.5043	–	0.8719	–
MOS21	0.6791	–	0.7765	–	0.6054	–	0.8958	–
UM20	0.4966	–	1.0376	–	0.5134	–	1.2626	–
UM21	0.6063	–	1.6095	–	0.4908	–	1.2180	–
Across	0.5377	0.0416	1.4438	0.2131	0.4534	0.1076	1.1472	0.0911

The largest ρ in bold font.

*HEL* Helgegården, *MOS* Mosslunda, *UM* Umeå, *20* Year 2020, *21* Year 2021, *Global* across six environments.

### Total tuber weight

Table 1 and Fig. 1A and B show the genomic prediction accuracy based on correlation, and NRMSE for ST-PLS and MT-PLS based on 5FCV and LOEO for each of the six location-year combinations (H20 H21, M20, M21, U20, U21) and across (global) environments.

#### Correlations

Results show that for 5FCV, MT-PLS gave slightly higher prediction accuracy than ST-PLS in terms of correlation (ρ). Exceptions were at the prediction of environments H21 (ST-PLS = 0.5761 vs MT-PLS = 0.5592) and U21 (ST-PLS = 0.5471 vs MT-PLS = 0.5260) obtained from 5FCV prediction (Fig. 1A 5FCV). Results across (global) for 5FCV show a correlation of 0.8017 for MT-PLS versus 0.7946 for ST-PLS.

Similar correlation results for LOEO show MT-PLS giving higher prediction accuracy than ST-PLS. Exceptions were at the prediction of environments M20 (ST-PLS = 0.6590 vs MT-PLS = 0.6523) and U21 (ST-PLS = 0.5548 vs MT-PLS = 0.5279) (Fig. 1A LOEO). Results across (global) for LOEO show a correlation of 0.6736 for MT-PLS versus 0.6656 for ST-PLS.

#### NRMSE

For the prediction accuracy measures by the NRMSE (Fig. 1B) the results were the same as those obtained for 5FCV. Note that for this matric the smaller the better. Results show that the MT-PLS gave higher prediction accuracy than ST-PLS in terms of NRMSE. Exceptions were at the prediction of environments H21 (ST-PLS = 0.8253 vs MT-PLS = 0.8417) and U21 (ST-PLS = 0.9098 vs MT-PLS = 0.9526) obtained from NRMSE. Results across (global) for 5FCV show a value 0.6000 for MT-PLS versus 0.6099 for ST-PLS.

Lower NRMS values for LOEO show MT-PLS giving higher prediction accuracy than ST-PLS. Exceptions were at the prediction of environments H21 (ST-PLS = 1.7354 vs MT-PLS = 1.7359) and M20 (ST-PLS = 0.8050 vs MT-PLS = 0.8151) (Fig. 1A LOEO). Results across (global) for LOEO show a value of 1.2061 for ST-PLS versus 1.1967 for MT-PLS. Note that results from LOEO cross-validation give slightly different results then those obtained from 5FCV.

### Flesh tuber starch

The genomic prediction accuracy measured based on correlation, and NRMSE for ST-PLS and MT-PLS for two metrics, 5FCV and LOEO, for each of the six location-year combination (H20 H21, M20, M21, U20, U21) and across all environments are given in Table 2 and Fig. 2A and B.

#### Correlations

In general, genomic predictions measured by correlations of trait flesh tuber starch are high (around 0.8–0.9) for most of the environments except for U21 (around 0.4). Results show that for 5FCV, MT-PLS gave higher prediction accuracy than ST-PLS in terms of correlation (ρ). For two environments results were different. M20 (ST-PLS = 0.8152 vs MT-PLS = 0.8126) and M21 (ST-PLS = 0.8012 vs MT-PLS = 0.7932) obtained from 5FCV prediction (Fig. 2A 5FCV). Results across (global) for 5FCV show a correlation of 0.9413 for MT-PLS versus 0.9367 for ST-PLS.

For LOEO cross-validations results show MT-PLS giving higher prediction accuracy than ST-PLS except for environment U21 (ST-PLS = 0.4831 vs MT-PLS = 0.4699) (Fig. 2A LOEO). Results across (global) for LOEO show a correlation of 0.9413 for MT-PLS versus 0.9367 for ST-PLS.

#### NRMSE

For the prediction accuracy measures by the NRMSE (Fig. 2B) results show that the MT-PLS gave higher prediction accuracy than ST-PLS in terms of NRMSE. One exception was the prediction of environments U21 (ST-PLS = 0.8789 vs MT-PLS = 0.8985). Results across (global) for 5FCV show a value 0.3433 for MT-PLS versus 0.3502 for ST-PLS.

Lower NRMS values for LOEO show MT-PLS giving higher prediction accuracy than ST-PLS. Exception was at the prediction of environments M21 (ST-PLS = 1.1575 vs MT-PLS = 1.1589) (Fig. 2A LOEO). Results across (global) for LOEO show a value of 1.6381 for ST-PLS versus 1.6319 for MT-PLS. As previously mentioned, results from LOEO cross-validation are slightly different than those obtained from 5FCV.

In summary for most of the environment’s MT-PLS gave higher prediction accuracy than ST-PLS for both correlation and NRMSE. Furthermore, results for this trait demonstrated the high prediction accuracy achieved for any metric used or any model including ST or MT but with a consistent increase of MT over ST for most of the environments.

### Flesh reducing sugar

Table 3 and Fig. 3A and B had the genomic prediction accuracy based on correlation, and NRMSE for ST-PLS and MT-PLS based on 5FCV and LOEO for each of the six location-year combinations (H20 H21, M20, M21, U20, U21) and across (global) environments.

#### Correlations

The correlations obtained by the 5FCV show that the MT-PLS are higher than those obtained by ST-PLS for each and across environments except for H20 (ST-PLS = 0.4247 vs MT-PLS = 0.3635 (Fig. 3A). However, correlation results from LOEO indicated that ST-PLS on prediction accuracy in H21 (ST-PLS = 0.5811 vs MT-PLS = -0.0624), M21 (ST-PLS = 0.6591 vs MT-PLS = 0.6054), U21 (ST-PLS = 0.6063 vs MT-PLS = 0.4908) and across environments (ST-PLS = 0.5377 vs MT-PLS = 0.4534) was larger than respective MT PLS, while surprising MT-PLS gave a zero prediction (or negative result) in H21.

#### NRMSE

In terms of judging the prediction accuracy of ST and MT based on NRMSE the results were not well defined as noticed for the other two traits. For criterion 5FCV, environments H20 (ST-PLS = 0.9451vs MT-PLS = 0.9896), U20 (ST-PLS = 0.9805 vs MT-PLS = 0.9950) and U21 (ST-PLS = 0.8194 vs MT-PLS = 0.8196) gave better predictions for ST-PLS than that obtained for MT-PLS. For LOEO criterion MT-PLS gave lower prediction accuracy than ST-PLS for H21 (ST-PLS = 1.2577 vs MT-PLS = 1.4475), M21 (ST-PLS = 0.7765 vs MT-PLS = 0.8958), and U20 (ST-PLS = 1.0376 vs MT-PLS = 1.2626) (Table 3 and Fig. 3B).

### Prediction accuracy for weight according to tuber size

The correlations between observed and predicted values for tuber weight at different sizes are given in Supplementary Figs. S1–S4, and in Supplementary Tables S1–S4. On average, the PLS-based prediction for all weights as per their tuber size were smaller than those noted for total tuber yield and tuber flesh starch. The largest prediction accuracy was noted for weight of tubers below 40 mm (Supplementary Fig. S1) or above 60 mm (Supplementary Fig. S4). The correlations obtained by the 5FCV were mostly higher than those obtained by LOEO. The MT-PLS prediction accuracy across environments was larger than the ST-PLS, though MT-PLS prediction accuracy in some environments was smaller than respective ST-PLS, e.g. for weight of tuber below 40 mm (H20), or for weight of tubers between 40 and 50 mm (M20). ST-PLS and MT-PLS gave a zero prediction (or negative result) for weight of tubers between 50 and 60 mm in H21.

## Discussion

Tuber flesh starch had the largest prediction accuracy (MT-PLS: 0.9416 ± 0.0045; ST-PLS: 0.9367 ± 0.052) under 5FCV (Fig. 2). This trait has the highest heritability (0.933) in the reference germplasm across the target population of environments of Scandinavia^29^. The prediction accuracy for total tuber weight (Fig. 1) was larger than those observed according to size (Figs. S1–S4). As indicated by Ortiz et al.^29^ total tuber weight also had larger heritability estimates (0.836) than those noted for tuber weight at different sizes (0.581–0.806). Reducing sugars in the tuber flesh had a lower prediction accuracy (Fig. 3) than both tuber flesh starch (Fig. 2) and total tuber weight (Fig. 1), which could result from having a smaller heritability (0.778) than the other two tuber traits^29^. These results suggest that applying selection based on genomic-estimated breeding values will be effective for high heritability traits in potato.

To the best of our knowledge the MT-PLS prediction accuracy for tuber flesh starch seems to be the largest ever estimated for any characteristic in potato. As per previous research, prediction accuracy for tuber flesh starch or specific gravity ranged from 0.09 to 0.83^20^. Similarly, the MT-PLS for tuber weight and reducing sugars in the tuber flesh are above or in the high end than those noted in early research, whose ranges were 0.05–0.75 and 0.11–0.79, respectively^20^. Most of these previous prediction accuracy estimates were based mostly of ST GBLUP. The ST models are trained to predict a single trait at a time (continuous, binary, categorical or count), while MT models are trained to simultaneously predict at least two traits. MT models are preferred over ST models because they represent complex relationships between traits, and simultaneously make use of the correlations between cultivar, traits, and environments. MT are more efficient to train computationally than each ST model, they improve indirect selection because of increased precision of genetic correlation estimates between traits. MT models can increase prediction accuracy of low heritability traits that have a significant correlation with high heritability^24,30^. MT models improve parameter estimates and prediction accuracy as compared to ST models if traits are moderately correlated^24,30–34^.

Adding multiple traits and multiple environments when using the PLS method for genomic prediction gives potato breeders more information that allows handling the significant genotype-by-environment interaction (GEI) that often affects tuber characteristics, particularly for total tuber yield. Prediction accuracy increases by considering GEI and correlated characteristics, thus improving the genomic selection approach for potato breeding in the target population of environments. Identifying breeding clones or cultivars according to their genomic estimated breeding values determined using PLS models that consider GEI will facilitate their further use as potential parents in potato breeding programs, thus increasing genetic gains.

The PLS method can be an alternative method for genomic prediction because it is very powerful for modelling data with inputs with large dimensionality and highly correlated; i.e., PLS naturally is able to handle more independent variables than observations that are highly correlated. PLS is the method for making good predictions in multivariate problems. Likewise, the PLS method offer high computational and statistical efficiency, as well as great flexibility and versatility in terms of the analysis problems that may be addressed^35^. For this reason the PLS method had been implemented in many areas of research for solving association and prediction problems^22,24,28,36,37^. In the case of prediction problems had been used for ST and MT predictions as well for the prediction of continuous, binary and categorical response variables. PLS originally was not proposed for association research, since the goal of the method was to find the significant linear subspace of the independent variables, not the variables themselves, but a large number of association research had been done applying PLS for variable selection. In this context, it has been used for the identification of genes associated with the considered outcome and for genome wide association study (GWAS) due to its competitive power and false discovery rates^38^.

## Conclusion

The PLS method is highly suited for genomic prediction in potato breeding when high dimensional and correlated genomic and other omics data are available. However, there were not large differences observed under a ST and MT framework. Likewise, better prediction performance was obtained under the prediction problem of tested lines and tested environments (5FCV), than under the tested line and untested environments (LOEO), which was expected because the LOEO cross-validation is a difficult prediction problem. The results are very promising since one can predict most potato traits with high accuracy using the PLS framework.

## Materials and methods

Multi-site testing involves six trials that included up to 256 breeding clones and released cultivars grown in Europe (https://hdl.handle.net/11529/10548617). The trials were held at Helgegården [HEL], Mosslunda [MOS] and Umeå [UM]) in 2020 and 2021 using simple lattices of 10-plant plots. The combination of location and years were denoted as environments such that six environments were included, H20, H21, M20, M21, U20, and U21).

HEL and MOS are at potato producing sites near Kristianstad (56°01′46″N 14°09′24″E) in Skåne, while Umeå (63°49′30″N 20°15′50″E) is in Norrland. The time between planting and harvest was between 3.5 to 4 months in Skåne, and about 90 days in Umeå. The temperatures were from 12 to 18 °C, and 12.5 to 16 °C in Skåne and Umeå, respectively, while the rainfall ranges were 42–64 mm in Skåne and 48–75 mm in Umeå. The average daylength ranged from 11.5 h (around harvest) to 17.5 h (mid-growing season) in Skåne, and from 14.5 (harvest) to ca. 21 h (early cropping season) in Umeå. Fungicides were used against the oomycete *Phytophthora infestans* in Helgegården to avoid late blight in the potato crop throughout the growing season. In this way, tuber yield potential could be estimated at this testing site. Tubers used as planting material were either from SLU’s Svensk potatisförädling or acquired through purchasing.

Relevant institutional, national, and international guidelines and legislation were considered for field research. Crop husbandry at each site was the same used for potato farming. The characteristics evaluated were total tuber yield in a 10-plant plot (kg), tuber weight (kg) by size (< 40 mm, 40–50 mm, 50–60 mm, > 60 mm) in the 10-plant plot, while tuber flesh starch was calculated by determining specific gravity after harvest^39^. Potato glucose strip tests were used for measuring reducing sugars in the tuber flesh^40^. Heritability based on variance components, as well as genetic and phenotypic correlations (Supplementary Tables S1 and S2) were estimated following Ortiz et al.^29^ Targeted genotyping –following a genotype-by-sequencing approach (https://www.diversityarrays.com/technology-and-resources/targeted-genotyping/) was used for characterizing 256 breeding clones and released cultivars with 2503 single nucleotide polymorphisms (SNPs), which were mostly derived after filtering SolCAP SNPs with known chromosome positions and MAF above 1% in germplasm from the Centro Internacional de la Papa (CIP, Lima, Perú) and the USA. Such a number of SNP suffices for genomic estimated breeding values without losing information^41^. The breeding clone 97 and cultivars ‘Leyla’ and ‘Red Lady’ were not included further in the genomic prediction analysis because they were lacking enough SNP data.

### Single-trait partial least squares (ST-PLS) and multi-trait partial least square (MT-PLS) methods

PLS is a single-trait (ST) and multi-trait (MT) regression statistical machine learning technique introduced by Wold^42^ in econometrics and chemometrics. PLS is very effective for prediction problems where the number of inputs ($$p)$$ is larger than the number of observations ($$n)$$; i.e., under $$p>n$$ problems, and also when inputs are highly correlated. This article describes the MT version of PLS, since the ST version works in a similar fashion to the MT version, except that the response variable $$(\mathbf{Y})$$ is a vector instead of a matrix. We assumed that we had a matrix of response variables $$(\mathbf{Y})$$ of order $$n\times {n}_{T}$$ ($${n}_{T}=\mathrm{number of traits}$$ that is related to a set of explanatory variables ($$\mathbf{X}$$) of order $$n\times p$$^35,43^. In PLS, instead of regressing $$\mathbf{Y}$$ on $$\mathbf{X}$$**,** we regressed $$\mathbf{Y}$$ on $$\mathbf{T}$$, where $$\mathbf{T}$$ are the latent variables (LVs), also called latent vectors or $$\mathbf{X}$$**-**scores; these LVs are related to the original $$\mathbf{X}$$ and $$\mathbf{Y}$$ matrices. The goal of PLS regression is to maximize the covariance between $$\mathbf{Y}$$ and $$\mathbf{T}$$; however, an iterative procedure is required for its computation. The basic steps to compute the LVs under a multivariate framework using the kernel algorithm for PLS are provided below.

*Step 1*. Initialization of matrices, $\mathbf{E}$ = $\mathbf{X}$ and $\mathbf{F}$ = $\mathbf{Y}$. Center each column of $\mathbf{E}$ and $\mathbf{F}$; scaling is optional.

*Step 2*. Compute $\mathbf{S}={\mathbf{X}}^{\mathrm{T}}\mathbf{Y}$ (Cross product matrix) and then $\mathbf{S}{\mathbf{S}}^{\mathrm{T}}={\mathbf{X}}^{\mathrm{T}}\mathbf{Y}{\mathbf{Y}}^{\mathrm{T}}\mathbf{X}$ and ${\mathbf{S}}^{\mathrm{T}}\mathbf{S}={\mathbf{Y}}^{\mathrm{T}}\mathbf{X}{\mathbf{X}}^{\mathrm{T}}\mathbf{Y}$**.**

*Step 3.* Compute the singular value decomposition (SVD) of $\mathbf{S}{\mathbf{S}}^{\mathrm{T}}$ and ${\mathbf{S}}^{\mathrm{T}}\mathbf{S}$.

Step *4.* Obtain $w$ and $q$**,** the eigenvectors to the largest eigenvalue of $\mathbf{S}{\mathbf{S}}^{\mathrm{T}}$ and ${\mathbf{S}}^{\mathrm{T}}\mathbf{S}$**,** respectively**.**

*Step 5.* Compute scores ${\varvec{t}}$ and ${\varvec{u}}$ as ${\varvec{t}}=\mathbf{X}{\varvec{w}}=\mathbf{E}{\varvec{w}}$ and ${\varvec{u}}=\mathbf{Y}{\varvec{q}}=\mathbf{F}{\varvec{q}}$.

*Step 6.* Normalize the ${\varvec{t}}$ and ${\varvec{u}}$ scores as ${\varvec{t}}={\varvec{t}}/\sqrt{{{\varvec{t}}}^{{\varvec{T}}}{\varvec{t}}}$ and ${\varvec{u}}={\varvec{u}}/\sqrt{{{\varvec{u}}}^{{\varvec{T}}}{\varvec{u}}}$.

*Step 7*. Next, compute $\mathbf{X}$ and $\mathbf{Y}$ loadings as ${\varvec{p}}={\mathbf{E}}^{{\varvec{T}}}{\varvec{t}}$ and ${\varvec{q}}={\mathbf{F}}^{{\varvec{T}}}{\varvec{t}}$**.**

*Step 8*. Deflate matrices $\mathbf{E}$ and $\mathbf{F}$ as ${{\varvec{E}}}_{n+1}={{\varvec{E}}}_{n}- {\varvec{t}}{{\varvec{p}}}^{{\varvec{T}}}$ and ${{\varvec{F}}}_{n+1}={{\varvec{F}}}_{n}- {\varvec{t}}{{\varvec{q}}}^{{\varvec{T}}}.$

*Step 9*. Use as input ${{\varvec{E}}}_{n+1}$ and ${{\varvec{F}}}_{n+1}$, of Step 8, in Step 2, and repeat steps 2 to 9 until the deflated matrices are empty or the necessary number of components have been extracted.

With the resulting ${\varvec{w}}$, ${\varvec{t}}$, ${\varvec{p}}$ and ${\varvec{q}}$ vectors, the matrices **W**, **T**, **P**, and **Q**, respectively, are built. Finally, after having all the columns of $\mathbf{W}$**,** we compute $\mathbf{R}$ as:

$$\mathbf{R}=\mathbf{W}{({\mathbf{P}}^{T}{\varvec{W}})}^{-1}$$

Next, with $\mathbf{R}$ we can compute the LVs, which are related to the original $\mathbf{X}$ matrix as:

$$\mathbf{T}=\mathbf{X}{\varvec{R}}$$

Next, since we regressed $\mathbf{Y}$ on $\mathbf{T}$, the resulting beta coefficients are $\mathbf{b}={({\mathbf{T}}^{T}{\varvec{T}})}^{-1}{\mathbf{T}}^{T}\mathbf{Y}$. However, to convert these back to the realm of the original variables (${\varvec{X}})$, we pre-multiplied with matrix ${\varvec{R}}$ the beta coefficients ($\mathbf{b}$); since $\mathbf{T}=\mathbf{X}{\varvec{R}},$

$$\mathbf{B}=\mathbf{R} \mathbf{b}$$

To obtain optimal performance of the PLS method, only the first $a$ components are used. Since regression and dimension reduction are performed simultaneously, all $\mathbf{B}$, $\mathbf{T}$, $\mathbf{W}$, $\mathbf{P}$ and $\mathbf{Q}$ are part of the output. Both $\mathbf{X}$ and $\mathbf{Y}$ are considered when calculating the LVs in ${\varvec{T}}$. Thereafter, predictions for new data (${{\varvec{X}}}_{{\varvec{n}}{\varvec{e}}{\varvec{w}}}$) should be done with:

$${\widehat{{\varvec{Y}}}}_{{\varvec{n}}{\varvec{e}}{\varvec{w}}}={\mathbf{X}}_{{\varvec{n}}{\varvec{e}}{\varvec{w}}}\mathbf{B}={{\varvec{X}}}_{{\varvec{n}}{\varvec{e}}{\varvec{w}}}\mathbf{R}\mathbf{b}={{\varvec{T}}}_{{\varvec{n}}{\varvec{e}}{\varvec{w}}}\mathbf{b}$$

where ${{\mathbf{T}}_{{\varvec{n}}{\varvec{e}}{\varvec{w}}}=\mathbf{X}}_{{\varvec{n}}{\varvec{e}}{\varvec{w}}}\mathbf{R}$. In this application, the optimal number of components was determined by cross-validation. We used the NRMSE, with an inner fivefold cross-validation for selecting the optimal number of hyperparameters.

In this application, we used as the input matrix or matrix of independent variables **X**, the concatenation of information of Environments + Genotypes + Genotypes $\times$ Environments information. For this reason, we first computed the design matrices of environments (${\mathbf{X}}_{\mathrm{E}}),$ the design matrix of genotypes (${\mathbf{X}}_{\mathrm{g}})$ and the design matrix of the Genotype $\times$ Environments term (${\mathbf{X}}_{\mathrm{gE}}$). Note that PLS method does not allow including directly (as mixed models do), genomic relationship information and genotype $\times$ environment interaction: (1) genomic relationship matrix of lines ${{\varvec{K}}}_{{\varvec{L}}}={\varvec{M}}{{\varvec{M}}}^{T}/r$ where ${\varvec{M}}$ denotes the matrix of markers (coded as 0, 1 and 2) of order $J\times r$, $J$ denotes the number of lines and $r$ the total number of markers, and the (2) genotype by environment relationship matrix (${{\varvec{K}}}_{{\varvec{L}}{\varvec{E}}}={{\varvec{K}}}_{{\varvec{E}}}\times {\mathbf{K}}_{\mathrm{L}})$, where $({{\varvec{K}}}_{{\varvec{E}}}={\mathbf{X}}_{\mathrm{E}}{\mathbf{X}}_{\mathrm{E}}^{T}/I)$ (where $I$ denotes the number of environments under study). Thus, to incorporate into the input matrix these relationship information’s, the design matrices of lines and genotype $\times$ environments were post-multiplied by their corresponding square root matrices of their corresponding relationship matrices. That is, instead of using only ${\mathbf{X}}_{\mathrm{g}}$ and ${\mathbf{X}}_{\mathrm{gE}}$ as input, we used ${\mathbf{X}}_{\mathrm{g}}{\mathbf{L}}_{\mathrm{g}}$ **(**with ${\mathbf{L}}_{\mathrm{g}}={{\varvec{K}}}_{L}^{0.5})\boldsymbol{ }\mathrm{and}$ ${\mathbf{X}}_{\mathrm{gE}}{\mathbf{L}}_{\mathbf{g}\mathbf{E}}$ (with ${\mathbf{L}}_{\mathrm{gE}}={{\varvec{K}}}_{LE}^{0.5})$**.** For this reason, the final input matrix used was $\mathbf{X}=\left[{\mathbf{X}}_{\mathrm{E}},{\mathbf{X}}_{\mathrm{g}}{\mathbf{L}}_{\mathbf{g}}, {\mathbf{X}}_{\mathrm{gE}}{\mathbf{L}}_{\mathrm{gE}}\right].$ We did not post-multiply the design matrix of environments (${\mathbf{X}}_{\mathrm{E}})$ since ${{\varvec{K}}}_{E}$ is an identity matrix due to the fact that we did not compute an environmental relationship matrix with environmental covariates, only with the dummy values of the position of environments. For this reason, both ST and MT PLS methods were used as input of the matrix of response variables (${\varvec{Y}})$ and the input matrix $\mathbf{X}$**,** just defined above, but the ST PLS was fitted one at a time for each column of ${\varvec{Y}}$**.** The implementation of both ST and MT PLS models was done with the R statistical software^44^ using the PLS^45^.

### Datasets and metrics for the evaluation of prediction accuracy

Answers to two prediction problems were pursued. The first was for tested lines in tested environments under a five-fold cross-validation (5FCV) strategy and the second was for tested lines in untested environments under a leave one environment out (LOEO) cross-validation strategy^46^. Under the 5FCV, we randomly divided the dataset into 5 subsets of similar size, using $5-1=4$ subsets as the outer training set and the remaining group as the outer testing set until each of the 5 subsets played the role of outer testing set once. Since we implemented PLS method (ST and MT), we divided the respective training set into an inner training set (80% of the training set) and a validation set (20% of the training set) to be able to tune (select the optimal) the number of principal components required in the PLS method. This nested cross-validation was also implemented under fivefold cross-validation. Then, the average of the five validation sets was reported as the accuracy of the predictions to select the optimal hyperparameter (principal components that must be retained). Then with this optimal hyperparameter we refitted the PLS method and with this refitted model we performed the prediction of each outer testing set. Again, prediction performance was reported as the average of the five outer testing sets.

Similarly, under the LOEO strategy of cross-validation, $I-1$ environments were assigned to the outer-training set and the remaining were assigned to the outer-testing set, until each of the $I$ environments were tested once. Also, for tuning the hyperparameter of the PLS (ST and MT) methods, we performed a nested 5FCV strategy, that is, the outer training set was five-fold, one was used as the validation set and the remaining four as inner-training. Then, the average of the five validation folds was reported as the metric of prediction performance to select the optimal hyperparameter (number of principal components) in the ST-PLS and MT-PLS models^44^. Then, using this optimal number of hyperparameters, both PLS models (ST and MT) were refitted with the whole training set (the $I-1$ environments) and finally, the prediction of each testing set (a full environment) was obtained. The 5FCV under inner and outer-cross-validation was repeated only one time. For the inner cross-validation under 5FCV and LOEO, we used as metric the normalized root mean square error ($NRMSE=\frac{RMSE}{\overline{y} }$), where $RMSE=\sqrt{\frac{1}{T}(\sum_{i=1}^{T}{({y}_{i}-\widehat{f}({x}_{i}))}^{2}}$, with ${y}_{i}$ denoting the observed value $i$, while $\widehat{f}({x}_{i})$ represents the predicted value for observation $i$, with $i=1,\dots ,n\mathrm{ number of observations})$. We used this metric for the inner cross-validation because it is one of the most appropriate metrics for comparisons when the model is multi-trait and the response variables are on different scales, since it is not dependent on the effect of the scale of the traits. However, for reporting the final accuracy (correlation between the predicted genetic value and the phenotypic value) with the outer cross-validation in addition to the NRMSE, we also reported the average Pearson’s Correlation.

## Supplementary Information

Supplementary Information.

## Data Availability

The genomic matrix ***K*** used in the models and all the data are stored at the link https://hdl.handle.net/11529/10548784, while the R scripts are available at: https://github.com/osval78/Potato_2023.
