# Supplementary material for: Partial least squares enhance multi-trait genomic prediction of potato cultivars in new environments
Source: Sci Rep. 2023 Jun 19;13:9947. doi: 10.1038/s41598-023-37169-y (PMC10279678; doi:10.1038/s41598-023-37169-y)
Supplement: Supplementary file 1 — Supplementary Information. [file 41598_2023_37169_MOESM1_ESM.docx]

**Partial least squares enhance multi-trait genomic prediction of potato cultivars in new environments**

Rodomiro Ortiz, Fredrik Reslow, Abelardo Montesinos-López, José Huicho, Paulino Pérez-Rodríguez, Osval A. Montesinos-López and José Crossa

**Supplementary Tables and Figures**

**Table S1**. Components of variance (σ^2^) and heritability (H^2^)for tuber weight (total and per size) and quality (starch and reducing sugars). All variance components were highly significant (*P* < 0.01).

| Statistic | Tuber weight (kg in 10-plant plot) | | | | | Flesh starch (%) | Reducing sugars |
| --- | --- | --- | --- | --- | --- | --- | --- |
|  | < 40 mm | 40–50 mm | 50–60 mm | > 60 mm | Total |  |  |
| H^2 z^ | 0.8391 | 0.7165 | 0.6793 | 0.8195 | 0.8645 | 0.9248 | 0.8176 |
| σ^2^_Genetic (G)_ | 0.2022 | 0.3633 | 0.5769 | 3.1980 | 5.2972 | 3.9326 | 0.3382 |
| σ^2^_GE_ | 0,1789 | 0.6135 | 1.2034 | 2.8232 | 3.3264 | 1.4322 | 0,1731 |
| σ^2^_Environment (E)_ | 0,2841 | 0.3283 | 1.1150 | 6.9529 | 8.8193 | 18.2365 | 0.0378 |
| σ^2^_Residual (e)_ | 0.1074 | 0.4978 | 0.8617 | 2.8070 | 3.3089 | 0.9679 | 0.5592 |
| Mean | 0.8869 | 2.4738 | 2.8672 | 2.8737 | 9.0862 | 11.4816 | 2.2023 |

^z^ H^2^ = σ^2^_G/[_σ^2^_G + (_σ^2^_GE/# environments) + (_σ^2^_e/ # environments × # replicates)]_

**Table S2.** Genetic (above diagonal) and phenotypic correlations for tuber traits among breeding clones and cultivars (N = 256) across testing sites over years. Correlations in bold (i.e., above 0.1225) are significant (*P* < 0.05)

| Trait | Tuber weight (kg in 10-plant plot) | | | | | Flesh starch (%) | Reducing sugars |
| --- | --- | --- | --- | --- | --- | --- | --- |
|  | < 40 mm | 40–50 mm | 50–60 mm | > 60 mm | Total |  |  |
| < 40 mm |  | **0.1236** | -0.1007 | **-0.1825** | -0.1079 | 0.0441 | 0.0195 |
| 40–50 mm | **0.4507** |  | 0,0779 | -0.0335 | 0.0913 | 0.0086 | 0.0068 |
| 50–60 mm | **-0.4099** | **0.3977** |  | **0.1521** | **0.2077** | -0.0262 | 0.0091 |
| > 60 mm | **-0.6137** | **-0.2190** | **0.5422** |  | **0.2071** | -0.0249 | -0.0086 |
| Total | **-0.3105** | **0.3551** | **0.8398** | **0.8079** |  | -0.0309 | 0.0018 |
| Flesh starch | 0.1082 | 0.0749 | -0.0498 | -0.0878 | -0.0379 |  | 0.0189 |
| Reducing sugars | 0.0665 | 0.0388 | 0.0393 | 0.0012 | 0.0396 | 0.0717 |  |

| **Table S3**. Partial least square (PLS) accuracy measured as correlation between observed and predicted values (ρ), and normalized Root Mean Squared Error (NRMSE) with their respective standard errors (SE) for genomic prediction of 40mm or less tuber weight considering single trait PLS (ST-PLS)- and multi-trait PLS (MT-PLS) for each location within each year and across environments considering five-fold random cross-validations (5FCV) and leaving one environment out (LOEO). **The largest ρ in bold font.** | | | | | | | | | |
| --- | --- | --- | --- | --- | --- | --- | --- | --- | --- |
| Environment ^z^ | | ρ | S.E. | NRMSE | S.E. | ρ | S.E. | NRMSE | S.E. |
|  | | ST-PLS | | | | MT-PLS | | | |
| 5FCV | HEL20 | **0.8016** | 0.0316 | 0.7458 | 0.0983 | 0.7629 | 0.0338 | 0.7471 | 0.0719 |
|  | HEL21 | 0.6421 | 0.0496 | 1.0508 | 0.0615 | 0.6088 | 0.0342 | 1.0706 | 0.0501 |
|  | MOS20 | 0.7010 | 0.0427 | 0.7160 | 0.0437 | 0.7045 | 0.0525 | 0.7101 | 0.0531 |
|  | MOS21 | 0.7125 | 0.0258 | 0.8263 | 0.0377 | 0.7422 | 0.0110 | 0.7872 | 0.0389 |
|  | UM20 | 0.6589 | 0.0278 | 0.7763 | 0.0152 | 0.6512 | 0.0149 | 0.7855 | 0.0228 |
|  | UM21 | 0.5878 | 0.0386 | 0.8045 | 0.0261 | 0.6073 | 0.0443 | 0.7984 | 0.0339 |
|  | Across | 0.7697 | 0.0067 | 0.6427 | 0.0100 | 0.7820 | 0.0109 | 0.6321 | 0.0181 |
| LOEO | HEL20 | 0.7509 | − | 0.9255 | − | 0.7653 | − | 0.8612 | − |
|  | HEL21 | 0.6103 | − | 2.1682 | − | 0.5988 | − | 2.1032 | − |
|  | MOS20 | 0.7014 | − | 0.7401 | − | 0.7218 | − | 0.7341 | − |
|  | MOS21 | 0.7064 | − | 1.6436 | − | 0.743 | − | 1.6645 | − |
|  | UM20 | 0.6773 | − | 1.1035 | − | 0.6700 | − | 1.1202 | − |
|  | UM21 | 0.6096 | − | 1.2049 | − | 0.6612 | − | 1.1952 | − |
|  | Across | 0.6760 | 0.0230 | 1.2976 | 0.2140 | 0.6933 | 0.0251 | 1.2797 | 0.2107 |
| ^z^ HEL = Helgegården, MOS = Mosslunda, UM = Umeå; 20 = Year 2020, 21 = Year 2021, Global = across six environments | | | | | | | | | |

| **Table S4**. Partial least square (PLS) accuracy measured as correlation between observed and predicted values (ρ), and normalized Root Mean Squared Error (NRMSE) with their respective standard errors (SE) for genomic prediction of 40−50mm of tuber weight considering single trait PLS (ST-PLS)- and multi-trait PLS (MT-PLS) for each location within each year and across environments considering five-fold random cross-validations (5FCV) and leaving one environment out (LOEO). **The largest ρ in bold font.** | | | | | | | | | |
| --- | --- | --- | --- | --- | --- | --- | --- | --- | --- |
| Environment ^z^ | | ρ | S.E. | NRMSE | S.E. | ρ | S.E. | NRMSE | S.E. |
|  | | ST-PLS | | | | MT-PLS | | | |
| 5FCV | HEL20 | 0.6008 | 0.0854 | 0.7915 | 0.0533 | **0.6710** | 0.0253 | 0.7569 | 0.0205 |
|  | HEL21 | 0.5008 | 0.0300 | 0.8922 | 0.0247 | 0.5135 | 0.0524 | 0.9035 | 0.0392 |
|  | MOS20 | 0.5085 | 0.0513 | 0.8879 | 0.0492 | 0.4504 | 0.0462 | 0.9078 | 0.0317 |
|  | MOS21 | 0.5521 | 0.0560 | 0.8382 | 0.0360 | 0.5328 | 0.0602 | 0.8574 | 0.0495 |
|  | UM20 | 0.3969 | 0.0310 | 0.9490 | 0.0165 | 0.4730 | 0.0255 | 0.8973 | 0.0196 |
|  | UM21 | 0.3488 | 0.0631 | 0.9580 | 0.0370 | 0.4268 | 0.0587 | 0.9137 | 0.0395 |
|  | Across | 0.6098 | 0.0146 | 0.8017 | 0.0096 | 0.6387 | 0.0055 | 0.7804 | 0.0066 |
| LOEO | HEL20 | 0.5327 | − | 0.8935 | − | 0.5088 | − | 0.9082 | − |
|  | HEL21 | 0.3973 | − | 1.3075 | − | 0.3316 | − | 1.3394 | − |
|  | MOS20 | 0.5460 | − | 0.8421 | − | 0.5514 | − | 0.8356 | − |
|  | MOS21 | 0.6028 | − | 1.1614 | − | 0.5992 | − | 1.1556 | − |
|  | UM20 | 0.3191 | − | 1.0716 | − | 0.3586 | − | 1.0432 | − |
|  | UM21 | 0.2490 | − | 1.1900 | − | 0.3163 | − | 1.1606 | − |
|  | Across | 0.4412 | 0.0575 | 1.0777 | 0.0735 | 0.4443 | 0.0504 | 1.0738 | 0.0752 |
| ^z^ HEL = Helgegården, MOS = Mosslunda, UM = Umeå; 20 = Year 2020, 21 = Year 2021, Global = across six environments | | | | | | | | | |

| **Table S5**. Partial least square (PLS) accuracy measured as correlation between observed and predicted values (ρ), and normalized Root Mean Squared Error (NRMSE) with their respective standard errors (SE) for genomic prediction of 50−60mm of tuber weight considering single trait PLS (ST-PLS)- and multi-trait PLS (MT-PLS) for each location within each year and across environments considering five-fold random cross-validations (5FCV) and leaving one environment out (LOEO). **The largest ρ in bold font.** | | | | | | | | | |
| --- | --- | --- | --- | --- | --- | --- | --- | --- | --- |
| Environment ^z^ | | ρ | S.E. | NRMSE | S.E. | ρ | S.E. | NRMSE | S.E. |
|  | | ST-PLS | | | | MT-PLS | | | |
| 5FCV | HEL20 | 0.5665 | 0.0579 | 0.8309 | 0.0452 | 0.5916 | 0.0778 | 0.8015 | 0.0491 |
|  | HEL21 | 0.1370 | 0.0746 | 1.0514 | 0.0331 | 0.3015 | 0.0300 | 0.9934 | 0.0362 |
|  | MOS20 | 0.5194 | 0.0440 | 0.8526 | 0.0222 | 0.5310 | 0.0284 | 0.8514 | 0.0180 |
|  | MOS21 | 0.5450 | 0.0527 | 0.8612 | 0.0490 | 0.6160 | 0.0471 | 0.8021 | 0.0495 |
|  | UM20 | 0.5648 | 0.0484 | 0.8524 | 0.0416 | 0.5978 | 0.0499 | 0.8209 | 0.0455 |
|  | UM21 | 0.5303 | 0.0367 | 0.8855 | 0.0390 | 0.5351 | 0.0345 | 0.8884 | 0.0266 |
|  | Across | 0.6455 | 0.0073 | 0.7754 | 0.0088 | **0.6887** | 0.0109 | 0.7334 | 0.0127 |
| LOEO | HEL20 | 0.6489 | − | 1.1860 |  | 0.6691 |  | 1.1876 | − |
|  | HEL21 | - 0.1437 | − | 1.4793 | − | - 0.0624 | − | 1.4475 | − |
|  | MOS20 | 0.4349 | − | 0.9074 | − | 0.5043 | − | 0.8719 | − |
|  | MOS21 | 0.5822 | − | 0.9196 | − | 0.6054 | − | 0.8958 | − |
|  | UM20 | 0.5271 | − | 1.2528 | − | 0.5134 | − | 1.2626 | − |
|  | UM21 | 0.4964 | − | 1.2338 | − | 0.4908 | − | 1.2180 | − |
|  | Across | 0.4243 | 0.1174 | 1.1631 | 0.0891 | 0.4534 | 0.1070 | 1.1472 | 0.0911 |
| ^z^ HEL = Helgegården, MOS = Mosslunda, UM = Umeå; 20 = Year 2020, 21 = Year 2021, Global = across six environments | | | | | | | | | |

| **Table S6**. Partial least square (PLS) accuracy measured as correlation between observed and predicted values (ρ), and normalized Root Mean Squared Error (NRMSE) with their respective standard errors (SE) for genomic prediction of higher than 60mm of tuber weight considering single trait PLS (ST-PLS)- and multi-trait PLS (MT-PLS) for each location within each year and across environments considering five-fold random cross-validations (5FCV) and leaving one environment out (LOEO). **The largest ρ in bold font.** | | | | | | | | | |
| --- | --- | --- | --- | --- | --- | --- | --- | --- | --- |
| Environment ^z^ | | ρ | S.E. | NRMSE | S.E. | ρ | S.E. | NRMSE | S.E. |
|  | | ST-PLS | | | | MT-PLS | | | |
| 5FCV | HEL20 | 0.7964 | 0.0247 | 0.6062 | 0.0323 | 0.8167 | 0.0218 | 0.5892 | 0.0319 |
|  | HEL21 | 0.6783 | 0.0303 | 0.7585 | 0.0242 | 0.6673 | 0.0308 | 0.7548 | 0.0223 |
|  | MOS20 | 0.6463 | 0.0566 | 0.7503 | 0.0491 | 0.6795 | 0.0413 | 0.7287 | 0.0366 |
|  | MOS21 | 0.7388 | 0.0083 | 0.7013 | 0.0118 | 0.7422 | 0.0098 | 0.6892 | 0.0096 |
|  | UM20 | 0.6727 | 0.0427 | 1.2380 | 0.0671 | 0.6507 | 0.0441 | 1.1034 | 0.0882 |
|  | UM21 | 0.4962 | 0.0725 | 1.5174 | 0.1629 | 0.5103 | 0.0615 | 1.3637 | 0.1609 |
|  | Across | 0.7985 | 0.0141 | 0.6100 | 0.0169 | 0.8142 | 0.0111 | 0.5876 | 0.0123 |
| LOEO  LOEO | HEL20 | 0.7938 | − | 0.6496 | − | **0.8205** | − | 0.6436 | − |
|  | HEL21 | 0.6310 | − | 1.5324 | − | 0.6370 | − | 1.5324 | − |
|  | MOS20 | 0.6503 | − | 0.7933 | − | 0.6626 | − | 0.7901 | − |
|  | MOS21 | 0.7661 | − | 0.8432 | − | 0.7997 | − | 0.8006 | − |
|  | UM20 | 0.6814 | − | 2.8190 | − | 0.6973 | − | 2.7634 | − |
|  | UM21 | 0.5124 | − | 2.9855 | − | 0.5639 | − | 2.9119 | − |
|  | Across | 0.6725 | 0.0414 | 1.6038 | 0.4296 | 0.6968 | 0.0401 | 1.5737 | 0.4197 |
| ^z^ HEL = Helgegården, MOS = Mosslunda, UM = Umeå; 20 = Year 2020, 21 = Year 2021, Global = across six environments | | | | | | | | | |
|  |  |  |  |  |  |  |  |  |  |

**Figure S1**. Weight of tubers below 40 mm. Above: Correlation (Cor) between observed and predicted values for multi-trait (MT) and single trait (ST) for 5 fold cross-validation (5FCV) and leave-one-environment-out (LOEO) for each location-year combination (H = Helgegården, M = Mosslunda, U = Umeå; 20 = Year 2020, 21 = Year 2021, Global = across six environments). Below: Normalized Root Mean Squared Error (NRMSE) for MT and ST for 5FCV and LOEO for each location-year combination (H = Helgegården, M = Mosslunda, U = Umeå; 20 = Year 2020, 21 = Year 2021, Global = across six environments)

**** ****

**Figure S2**. Weight of tubers between 40 and 50 mm. Above: Correlation (Cor) between observed and predicted values for multi-trait (MT) and single trait (ST) for 5 fold cross-validation (5FCV) and leave-one-environment-out (LOEO) for each location-year combination (H = Helgegården, M = Mosslunda, U = Umeå; 20 = Year 2020, 21 = Year 2021, Global = across six environments). Below: Normalized Root Mean Squared Error (NRMSE) for MT and ST for 5FCV and LOEO for each location-year combination (H = Helgegården, M = Mosslunda, U = Umeå; 20 = Year 2020, 21 = Year 2021, Global = across six environments)

**Figure S3**. Weight of tubers between 50 and 60 mm. Above: Correlation (Cor) between observed and predicted values for multi-trait (MT) and single trait (ST) for 5 fold cross-validation (5FCV) and leave-one-environment-out (LOEO) for each location-year combination (H = Helgegården, M = Mosslunda, U = Umeå; 20 = Year 2020, 21 = Year 2021, Global = across six environments). Below: Normalized Root Mean Squared Error (NRMSE) for MT and ST for 5FCV and LOEO for each location-year combination (H = Helgegården, M = Mosslunda, U = Umeå; 20 = Year 2020, 21 = Year 2021, Global = across six environments.)

**Figure S4**. Weight of tubers above 60 mm. Above: Correlation (Cor) between observed and predicted values for multi-trait (MT) and single trait (ST) for 5 fold cross-validation (5FCV) and leave-one-environment-out (LOEO) for each location-year combination (H = Helgegården, M = Mosslunda, U = Umeå; 20 = Year 2020, 21 = Year 2021, Global = across six environments). Below: Normalized Root Mean Squared Error (NRMSE) for MT and ST for 5FCV and LOEO for each location-year combination (H = Helgegården, M = Mosslunda, U = Umeå; 20 = Year 2020, 21 = Year 2021, Global = across six environments)
